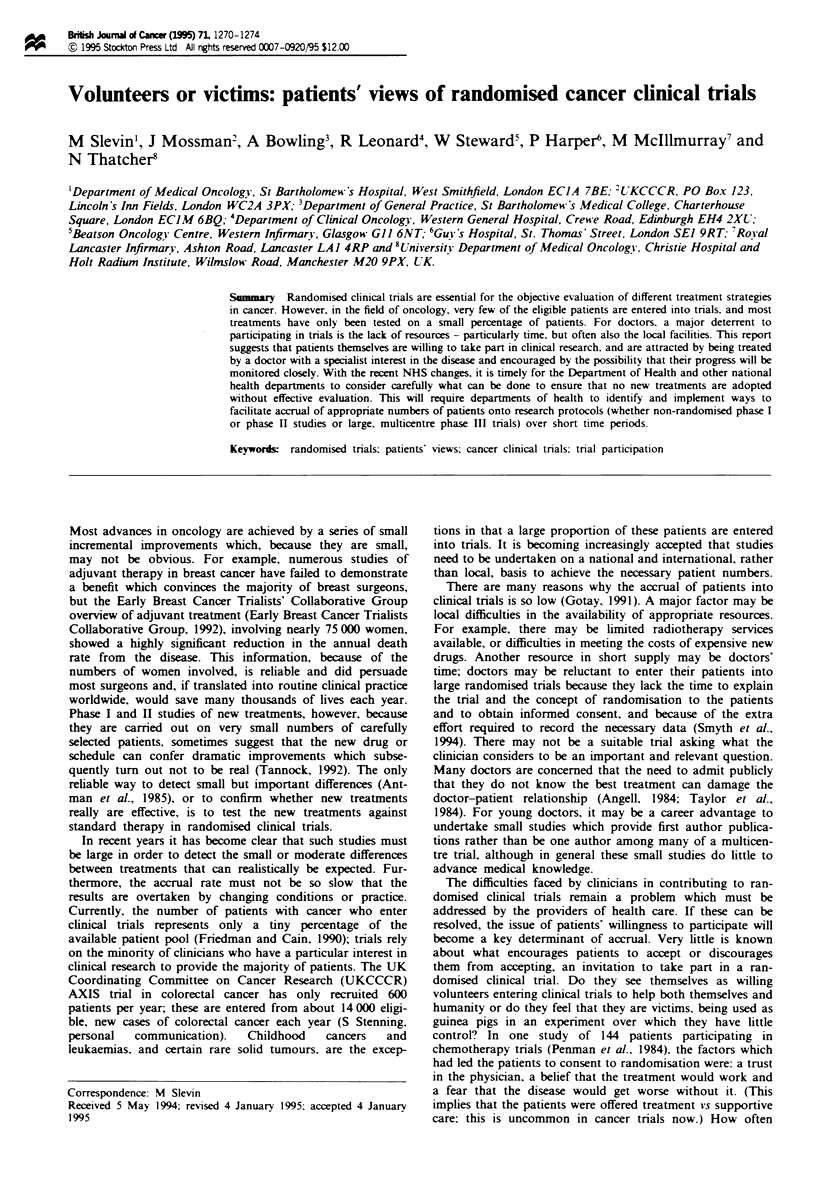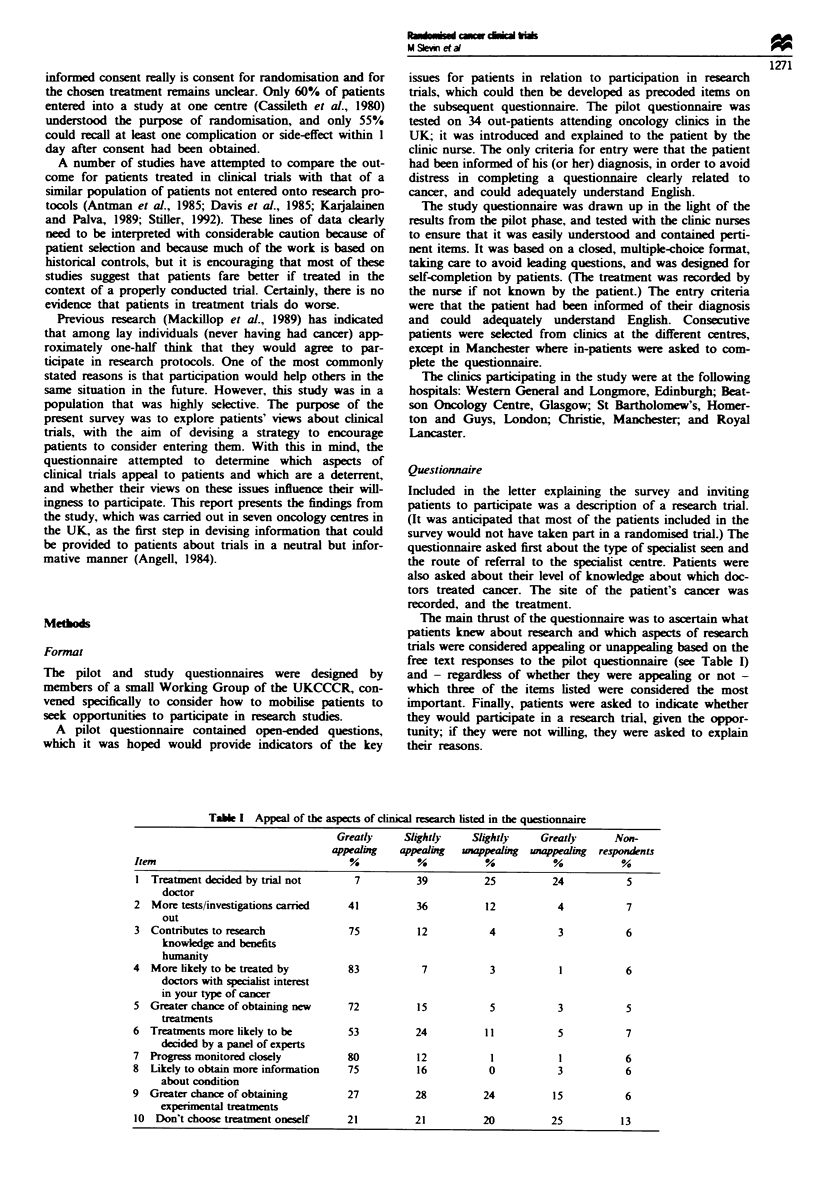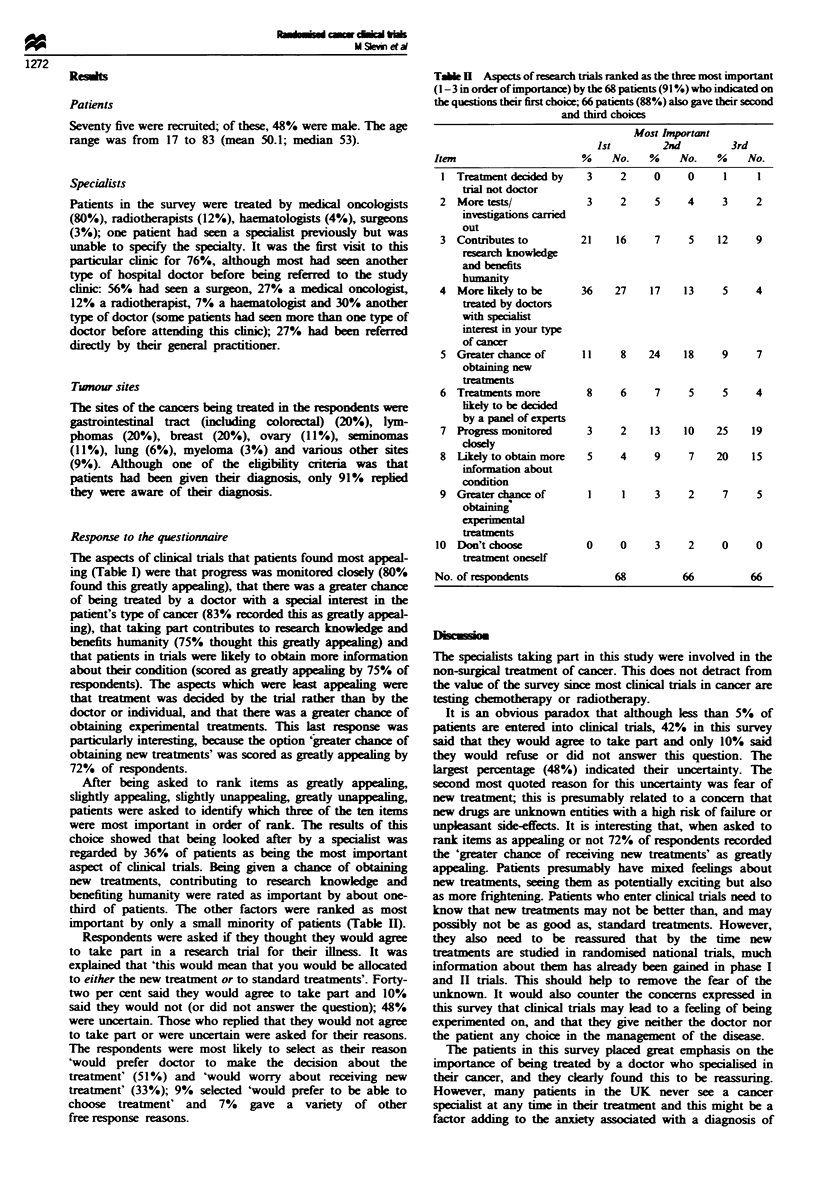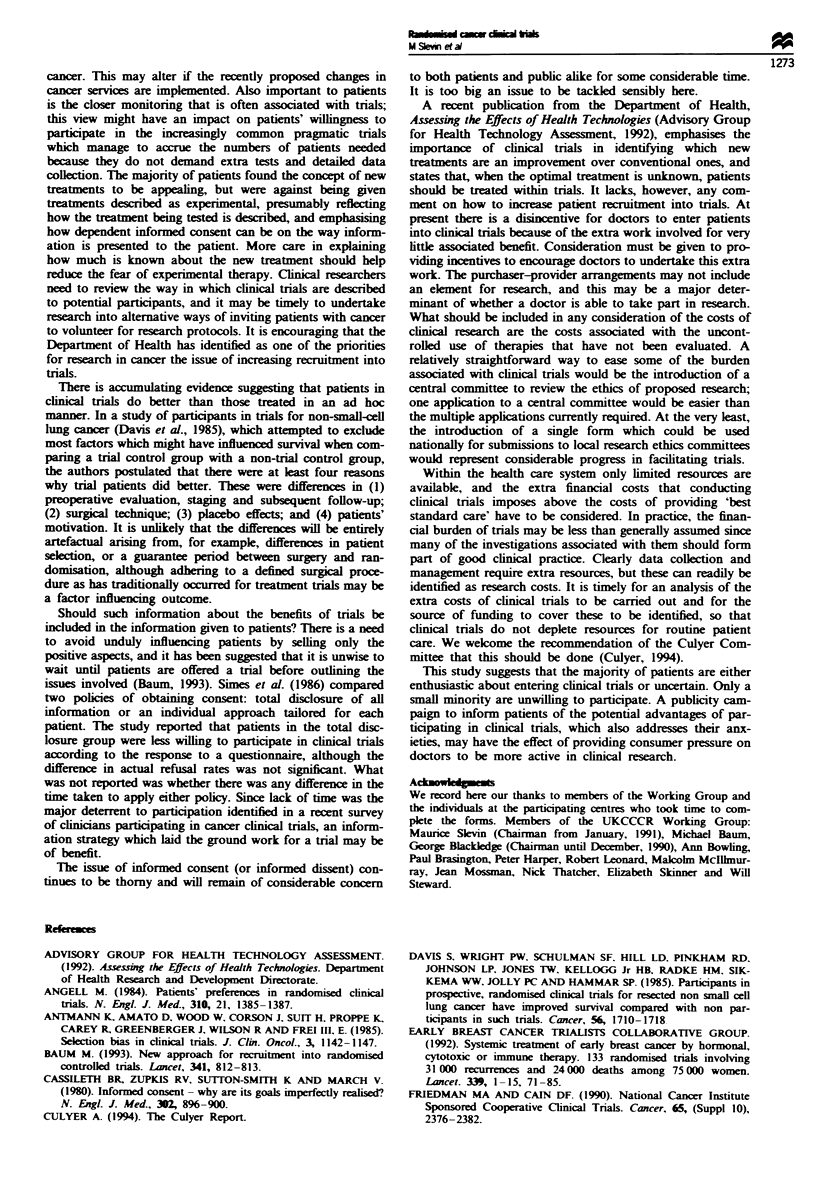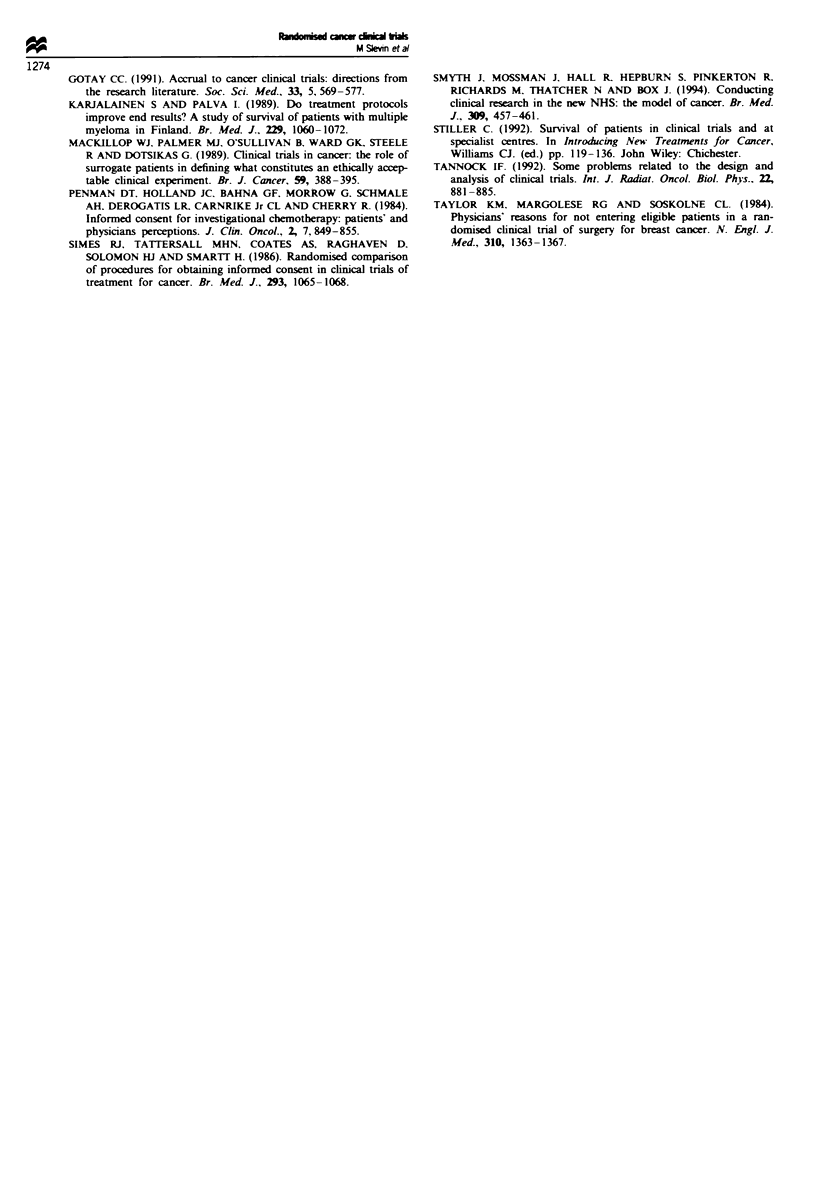# Volunteers or victims: patients' views of randomised cancer clinical trials.

**DOI:** 10.1038/bjc.1995.245

**Published:** 1995-06

**Authors:** M. Slevin, J. Mossman, A. Bowling, R. Leonard, W. Steward, P. Harper, M. McIllmurray, N. Thatcher

**Affiliations:** Department of Medical Oncology, St Bartholomew's Hospital, West Smithfield, London, UK.

## Abstract

Randomised clinical trials are essential for the objective evaluation of different treatment strategies in cancer. However, in the field of oncology, very few of the eligible patients are entered into trials, and most treatments have only been tested on a small percentage of patients. For doctors, a major deterrent to participating in trials is the lack of resources--particularly time, but often also the local facilities. This report suggests that patients themselves are willing to take part in clinical research, and are attracted by being treated by a doctor with a specialist interest in the disease and encouraged by the possibility that their progress will be monitored closely. With the recent NHS changes, it is timely for the Department of Health and other national health departments to consider carefully what can be done to ensure that no new treatments are adopted without effective evaluation. This will require departments of health to identify and implement ways to facilitate accrual of appropriate numbers of patients onto research protocols (whether non-randomised phase I or phase II studies or large, multicentre phase III trials) over short time periods.


### Volunteers or victims: patients' views of randomised cancer clinical trials

M Slevin[1], J Mossman[2], A Bowling[3], R Leonard[4], W Steward[5], P Harper[6], M McIllmurray[7] and N Thatcher[8]

[1]*Department of Medical Oncology, St Bartholomew's Hospital, West Smithfield, London EC1A 7BE;* [2]*UKCCCR, PO Box 123, Lincoln's Inn Fields, London WC2A 3PX;* [3]*Department of General Practice, St Bartholomew's Medical College, Charterhouse Square, London EC1M 6BQ;* [4]*Department of Clinical Oncology, Western General Hospital, Crewe Road, Edinburgh EH4 2XU;* [5]*Beatson Oncology Centre, Western Infirmary, Glasgow G11 6NT;* [6]*Guy's Hospital, St. Thomas' Street, London SE1 9RT;* [7]*Royal Lancaster Infirmary, Ashton Road, Lancaster LA1 4RP and* [8]*University Department of Medical Oncology, Christie Hospital and Holt Radium Institute, Wilmslow Road, Manchester M20 9PX, UK.*

**Summary** Randomised clinical trials are essential for the objective evaluation of different treatment strategies in cancer. However, in the field of oncology, very few of the eligible patients are entered into trials, and most treatments have only been tested on a small percentage of patients. For doctors, a major deterrent to participating in trials is the lack of resources – particularly time, but often also the local facilities. This report suggests that patients themselves are willing to take part in clinical research, and are attracted by being treated by a doctor with a specialist interest in the disease and encouraged by the possibility that their progress will be monitored closely. With the recent NHS changes, it is timely for the Department of Health and other national health departments to consider carefully what can be done to ensure that no new treatments are adopted without effective evaluation. This will require departments of health to identify and implement ways to facilitate accrual of appropriate numbers of patients onto research protocols (whether non-randomised phase I or phase II studies or large, multicentre phase III trials) over short time periods.

**Keywords:** randomised trials; patients' views; cancer clinical trials; trial participation

Most advances in oncology are achieved by a series of small incremental improvements which, because they are small, may not be obvious. For example, numerous studies of adjuvant therapy in breast cancer have failed to demonstrate a benefit which convinces the majority of breast surgeons, but the Early Breast Cancer Trialists' Collaborative Group overview of adjuvant treatment (Early Breast Cancer Trialists Collaborative Group, 1992), involving nearly 75 000 women, showed a highly significant reduction in the annual death rate from the disease. This information, because of the numbers of women involved, is reliable and did persuade most surgeons and, if translated into routine clinical practice worldwide, would save many thousands of lives each year. Phase I and II studies of new treatments, however, because they are carried out on very small numbers of carefully selected patients, sometimes suggest that the new drug or schedule can confer dramatic improvements which subsequently turn out not to be real (Tannock, 1992). The only reliable way to detect small but important differences (Antman *et al.*, 1985), or to confirm whether new treatments really are effective, is to test the new treatments against standard therapy in randomised clinical trials.

In recent years it has become clear that such studies must be large in order to detect the small or moderate differences between treatments that can realistically be expected. Furthermore, the accrual rate must not be so slow that the results are overtaken by changing conditions or practice. Currently, the number of patients with cancer who enter clinical trials represents only a tiny percentage of the available patient pool (Friedman and Cain, 1990); trials rely on the minority of clinicians who have a particular interest in clinical research to provide the majority of patients. The UK Coordinating Committee on Cancer Research (UKCCCR) AXIS trial in colorectal cancer has only recruited 600 patients per year; these are entered from about 14 000 eligible, new cases of colorectal cancer each year (S Stenning, personal communication). Childhood cancers and leukaemias, and certain rare solid tumours, are the exceptions in that a large proportion of these patients are entered into trials. It is becoming increasingly accepted that studies need to be undertaken on a national and international, rather than local, basis to achieve the necessary patient numbers.

There are many reasons why the accrual of patients into clinical trials is so low (Gotay, 1991). A major factor may be local difficulties in the availability of appropriate resources. For example, there may be limited radiotherapy services available, or difficulties in meeting the costs of expensive new drugs. Another resource in short supply may be doctors' time; doctors may be reluctant to enter their patients into large randomised trials because they lack the time to explain the trial and the concept of randomisation to the patients and to obtain informed consent, and because of the extra effort required to record the necessary data (Smyth *et al.*, 1994). There may not be a suitable trial asking what the clinician considers to be an important and relevant question. Many doctors are concerned that the need to admit publicly that they do not know the best treatment can damage the doctor–patient relationship (Angell, 1984; Taylor *et al.*, 1984). For young doctors, it may be a career advantage to undertake small studies which provide first author publications rather than be one author among many of a multicentre trial, although in general these small studies do little to advance medical knowledge.

The difficulties faced by clinicians in contributing to randomised clinical trials remain a problem which must be addressed by the providers of health care. If these can be resolved, the issue of patients' willingness to participate will become a key determinant of accrual. Very little is known about what encourages patients to accept or discourages them from accepting, an invitation to take part in a randomised clinical trial. Do they see themselves as willing volunteers entering clinical trials to help both themselves and humanity or do they feel that they are victims, being used as guinea pigs in an experiment over which they have little control? In one study of 144 patients participating in chemotherapy trials (Penman *et al.*, 1984), the factors which had led the patients to consent to randomisation were: a trust in the physician, a belief that the treatment would work and a fear that the disease would get worse without it. (This implies that the patients were offered treatment *vs* supportive care; this is uncommon in cancer trials now.) How often

Correspondence: M Slevin
Received 5 May 1994; revised 4 January 1995; accepted 4 January 1995

informed consent really is consent for randomisation and for the chosen treatment remains unclear. Only 60% of patients entered into a study at one centre (Cassileth *et al*., 1980) understood the purpose of randomisation, and only 55% could recall at least one complication or side-effect within 1 day after consent had been obtained.

A number of studies have attempted to compare the outcome for patients treated in clinical trials with that of a similar population of patients not entered onto research protocols (Antman *et al*., 1985; Davis *et al*., 1985; Karjalainen and Palva, 1989; Stiller, 1992). These lines of data clearly need to be interpreted with considerable caution because of patient selection and because much of the work is based on historical controls, but it is encouraging that most of these studies suggest that patients fare better if treated in the context of a properly conducted trial. Certainly, there is no evidence that patients in treatment trials do worse.

Previous research (Mackillop *et al*., 1989) has indicated that among lay individuals (never having had cancer) approximately one-half think that they would agree to participate in research protocols. One of the most commonly stated reasons is that participation would help others in the same situation in the future. However, this study was in a population that was highly selective. The purpose of the present survey was to explore patients' views about clinical trials, with the aim of devising a strategy to encourage patients to consider entering them. With this in mind, the questionnaire attempted to determine which aspects of clinical trials appeal to patients and which are a deterrent, and whether their views on these issues influence their willingness to participate. This report presents the findings from the study, which was carried out in seven oncology centres in the UK, as the first step in devising information that could be provided to patients about trials in a neutral but informative manner (Angell, 1984).

### Methods

### *Format*

The pilot and study questionnaires were designed by members of a small Working Group of the UKCCCR, convened specifically to consider how to mobilise patients to seek opportunities to participate in research studies.

A pilot questionnaire contained open-ended questions, which it was hoped would provide indicators of the key issues for patients in relation to participation in research trials, which could then be developed as precoded items on the subsequent questionnaire. The pilot questionnaire was tested on 34 out-patients attending oncology clinics in the UK; it was introduced and explained to the patient by the clinic nurse. The only criteria for entry were that the patient had been informed of his (or her) diagnosis, in order to avoid distress in completing a questionnaire clearly related to cancer, and could adequately understand English.

The study questionnaire was drawn up in the light of the results from the pilot phase, and tested with the clinic nurses to ensure that it was easily understood and contained pertinent items. It was based on a closed, multiple-choice format, taking care to avoid leading questions, and was designed for self-completion by patients. (The treatment was recorded by the nurse if not known by the patient.) The entry criteria were that the patient had been informed of their diagnosis and could adequately understand English. Consecutive patients were selected from clinics at the different centres, except in Manchester where in-patients were asked to complete the questionnaire.

The clinics participating in the study were at the following hospitals: Western General and Longmore, Edinburgh; Beatson Oncology Centre, Glasgow; St Bartholomew's, Homerton and Guys, London; Christie, Manchester; and Royal Lancaster.

### *Questionnaire*

Included in the letter explaining the survey and inviting patients to participate was a description of a research trial. (It was anticipated that most of the patients included in the survey would not have taken part in a randomised trial.) The questionnaire asked first about the type of specialist seen and the route of referral to the specialist centre. Patients were also asked about their level of knowledge about which doctors treated cancer. The site of the patient's cancer was recorded, and the treatment.

The main thrust of the questionnaire was to ascertain what patients knew about research and which aspects of research trials were considered appealing or unappealing based on the free text responses to the pilot questionnaire (see Table I) and – regardless of whether they were appealing or not – which three of the items listed were considered the most important. Finally, patients were asked to indicate whether they would participate in a research trial, given the opportunity; if they were not willing, they were asked to explain their reasons.

**Table I** Appeal of the aspects of clinical research listed in the questionnaire

| *Item* | *Greatly appealing* % | *Slightly appealing* % | *Slightly unappealing* % | *Greatly unappealing* % | *Non-respondents* % |
|---|---|---|---|---|---|
| 1 Treatment decided by trial not doctor | 7 | 39 | 25 | 24 | 5 |
| 2 More tests/investigations carried out | 41 | 36 | 12 | 4 | 7 |
| 3 Contributes to research knowledge and benefits humanity | 75 | 12 | 4 | 3 | 6 |
| 4 More likely to be treated by doctors with specialist interest in your type of cancer | 83 | 7 | 3 | 1 | 6 |
| 5 Greater chance of obtaining new treatments | 72 | 15 | 5 | 3 | 5 |
| 6 Treatments more likely to be decided by a panel of experts | 53 | 24 | 11 | 5 | 7 |
| 7 Progress monitored closely | 80 | 12 | 1 | 1 | 6 |
| 8 Likely to obtain more information about condition | 75 | 16 | 0 | 3 | 6 |
| 9 Greater chance of obtaining experimental treatments | 27 | 28 | 24 | 15 | 6 |
| 10 Don't choose treatment oneself | 21 | 21 | 20 | 25 | 13 |

### Results

### Patients

Seventy five were recruited; of these, 48% were male. The age range was from 17 to 83 (mean 50.1; median 53).

### Specialists

Patients in the survey were treated by medical oncologists (80%), radiotherapists (12%), haematologists (4%), surgeons (3%); one patient had seen a specialist previously but was unable to specify the specialty. It was the first visit to this particular clinic for 76%, although most had seen another type of hospital doctor before being referred to the study clinic: 56% had seen a surgeon, 27% a medical oncologist, 12% a radiotherapist, 7% a haematologist and 30% another type of doctor (some patients had seen more than one type of doctor before attending this clinic); 27% had been referred directly by their general practitioner.

### Tumour sites

The sites of the cancers being treated in the respondents were gastrointestinal tract (including colorectal) (20%), lymphomas (20%), breast (20%), ovary (11%), seminomas (11%), lung (6%), myeloma (3%) and various other sites (9%). Although one of the eligibility criteria was that patients had been given their diagnosis, only 91% replied they were aware of their diagnosis.

### Response to the questionnaire

The aspects of clinical trials that patients found most appealing (Table I) were that progress was monitored closely (80% found this greatly appealing), that there was a greater chance of being treated by a doctor with a special interest in the patient's type of cancer (83% recorded this as greatly appealing), that taking part contributes to research knowledge and benefits humanity (75% thought this greatly appealing) and that patients in trials were likely to obtain more information about their condition (scored as greatly appealing by 75% of respondents). The aspects which were least appealing were that treatment was decided by the trial rather than by the doctor or individual, and that there was a greater chance of obtaining experimental treatments. This last response was particularly interesting, because the option 'greater chance of obtaining new treatments' was scored as greatly appealing by 72% of respondents.

After being asked to rank items as greatly appealing, slightly appealing, slightly unappealing, greatly unappealing, patients were asked to identify which three of the ten items were most important in order of rank. The results of this choice showed that being looked after by a specialist was regarded by 36% of patients as being the most important aspect of clinical trials. Being given a chance of obtaining new treatments, contributing to research knowledge and benefiting humanity were rated as important by about one-third of patients. The other factors were ranked as most important by only a small minority of patients (Table II).

Respondents were asked if they thought they would agree to take part in a research trial for their illness. It was explained that 'this would mean that you would be allocated to *either* the new treatment *or* to standard treatments'. Forty-two per cent said they would agree to take part and 10% said they would not (or did not answer the question); 48% were uncertain. Those who replied that they would not agree to take part or were uncertain were asked for their reasons. The respondents were most likely to select as their reason 'would prefer doctor to make the decision about the treatment' (51%) and 'would worry about receiving new treatment' (33%); 9% selected 'would prefer to be able to choose treatment' and 7% gave a variety of other free response reasons.

**Table II** Aspects of research trials ranked as the three most important (1–3 in order of importance) by the 68 patients (91%) who indicated on the questions their first choice; 66 patients (88%) also gave their second and third choices

| | Most Important | | | | | |
|---|---|---|---|---|---|---|
| | 1st | | 2nd | | 3rd | |
| *Item* | % | No. | % | No. | % | No. |
| 1 Treatment decided by trial not doctor | 3 | 2 | 0 | 0 | 1 | 1 |
| 2 More tests/ investigations carried out | 3 | 2 | 5 | 4 | 3 | 2 |
| 3 Contributes to research knowledge and benefits humanity | 21 | 16 | 7 | 5 | 12 | 9 |
| 4 More likely to be treated by doctors with specialist interest in your type of cancer | 36 | 27 | 17 | 13 | 5 | 4 |
| 5 Greater chance of obtaining new treatments | 11 | 8 | 24 | 18 | 9 | 7 |
| 6 Treatments more likely to be decided by a panel of experts | 8 | 6 | 7 | 5 | 5 | 4 |
| 7 Progress monitored closely | 3 | 2 | 13 | 10 | 25 | 19 |
| 8 Likely to obtain more information about condition | 5 | 4 | 9 | 7 | 20 | 15 |
| 9 Greater chance of obtaining experimental treatments | 1 | 1 | 3 | 2 | 7 | 5 |
| 10 Don't choose treatment oneself | 0 | 0 | 3 | 2 | 0 | 0 |
| No. of respondents | | 68 | | 66 | | 66 |

### Discussion

The specialists taking part in this study were involved in the non-surgical treatment of cancer. This does not detract from the value of the survey since most clinical trials in cancer are testing chemotherapy or radiotherapy.

It is an obvious paradox that although less than 5% of patients are entered into clinical trials, 42% in this survey said that they would agree to take part and only 10% said they would refuse or did not answer this question. The largest percentage (48%) indicated their uncertainty. The second most quoted reason for this uncertainty was fear of new treatment; this is presumably related to a concern that new drugs are unknown entities with a high risk of failure or unpleasant side-effects. It is interesting that, when asked to rank items as appealing or not 72% of respondents recorded the 'greater chance of receiving new treatments' as greatly appealing. Patients presumably have mixed feelings about new treatments, seeing them as potentially exciting but also as more frightening. Patients who enter clinical trials need to know that new treatments may not be better than, and may possibly not be as good as, standard treatments. However, they also need to be reassured that by the time new treatments are studied in randomised national trials, much information about them has already been gained in phase I and II trials. This should help to remove the fear of the unknown. It would also counter the concerns expressed in this survey that clinical trials may lead to a feeling of being experimented on, and that they give neither the doctor nor the patient any choice in the management of the disease.

The patients in this survey placed great emphasis on the importance of being treated by a doctor who specialised in their cancer, and they clearly found this to be reassuring. However, many patients in the UK never see a cancer specialist at any time in their treatment and this might be a factor adding to the anxiety associated with a diagnosis of cancer. This may alter if the recently proposed changes in cancer services are implemented. Also important to patients is the closer monitoring that is often associated with trials; this view might have an impact on patients' willingness to participate in the increasingly common pragmatic trials which manage to accrue the numbers of patients needed because they do not demand extra tests and detailed data collection. The majority of patients found the concept of new treatments to be appealing, but were against being given treatments described as experimental, presumably reflecting how the treatment being tested is described, and emphasising how dependent informed consent can be on the way information is presented to the patient. More care in explaining how much is known about the new treatment should help reduce the fear of experimental therapy. Clinical researchers need to review the way in which clinical trials are described to potential participants, and it may be timely to undertake research into alternative ways of inviting patients with cancer to volunteer for research protocols. It is encouraging that the Department of Health has identified as one of the priorities for research in cancer the issue of increasing recruitment into trials.

There is accumulating evidence suggesting that patients in clinical trials do better than those treated in an ad hoc manner. In a study of participants in trials for non-small-cell lung cancer (Davis *et al.*, 1985), which attempted to exclude most factors which might have influenced survival when comparing a trial control group with a non-trial control group, the authors postulated that there were at least four reasons why trial patients did better. These were differences in (1) preoperative evaluation, staging and subsequent follow-up; (2) surgical technique; (3) placebo effects; and (4) patients' motivation. It is unlikely that the differences will be entirely artefactual arising from, for example, differences in patient selection, or a guarantee period between surgery and randomisation, although adhering to a defined surgical procedure as has traditionally occurred for treatment trials may be a factor influencing outcome.

Should such information about the benefits of trials be included in the information given to patients? There is a need to avoid unduly influencing patients by selling only the positive aspects, and it has been suggested that it is unwise to wait until patients are offered a trial before outlining the issues involved (Baum, 1993). Simes *et al.* (1986) compared two policies of obtaining consent: total disclosure of all information or an individual approach tailored for each patient. The study reported that patients in the total disclosure group were less willing to participate in clinical trials according to the response to a questionnaire, although the difference in actual refusal rates was not significant. What was not reported was whether there was any difference in the time taken to apply either policy. Since lack of time was the major deterrent to participation identified in a recent survey of clinicians participating in cancer clinical trials, an information strategy which laid the ground work for a trial may be of benefit.

The issue of informed consent (or informed dissent) continues to be thorny and will remain of considerable concern to both patients and public alike for some considerable time. It is too big an issue to be tackled sensibly here.

A recent publication from the Department of Health, *Assessing the Effects of Health Technologies* (Advisory Group for Health Technology Assessment, 1992), emphasises the importance of clinical trials in identifying which new treatments are an improvement over conventional ones, and states that, when the optimal treatment is unknown, patients should be treated within trials. It lacks, however, any comment on how to increase patient recruitment into trials. At present there is a disincentive for doctors to enter patients into clinical trials because of the extra work involved for very little associated benefit. Consideration must be given to providing incentives to encourage doctors to undertake this extra work. The purchaser–provider arrangements may not include an element for research, and this may be a major determinant of whether a doctor is able to take part in research. What should be included in any consideration of the costs of clinical research are the costs associated with the uncontrolled use of therapies that have not been evaluated. A relatively straightforward way to ease some of the burden associated with clinical trials would be the introduction of a central committee to review the ethics of proposed research; one application to a central committee would be easier than the multiple applications currently required. At the very least, the introduction of a single form which could be used nationally for submissions to local research ethics committees would represent considerable progress in facilitating trials.

Within the health care system only limited resources are available, and the extra financial costs that conducting clinical trials imposes above the costs of providing 'best standard care' have to be considered. In practice, the financial burden of trials may be less than generally assumed since many of the investigations associated with them should form part of good clinical practice. Clearly data collection and management require extra resources, but these can readily be identified as research costs. It is timely for an analysis of the extra costs of clinical trials to be carried out and for the source of funding to cover these to be identified, so that clinical trials do not deplete resources for routine patient care. We welcome the recommendation of the Culyer Committee that this should be done (Culyer, 1994).

This study suggests that the majority of patients are either enthusiastic about entering clinical trials or uncertain. Only a small minority are unwilling to participate. A publicity campaign to inform patients of the potential advantages of participating in clinical trials, which also addresses their anxieties, may have the effect of providing consumer pressure on doctors to be more active in clinical research.

**Acknowledgements**

We record here our thanks to members of the Working Group and the individuals at the participating centres who took time to complete the forms. Members of the UKCCCR Working Group: Maurice Slevin (Chairman from January, 1991), Michael Baum, George Blackledge (Chairman until December, 1990), Ann Bowling, Paul Brasington, Peter Harper, Robert Leonard, Malcolm McIllmurray, Jean Mossman, Nick Thatcher, Elizabeth Skinner and Will Steward.

### References

ADVISORY GROUP FOR HEALTH TECHNOLOGY ASSESSMENT. (1992). *Assessing the Effects of Health Technologies*. Department of Health Research and Development Directorate.

ANGELL M. (1984). Patients' preferences in randomised clinical trials. *N. Engl. J. Med.*, **310**, 21, 1385–1387.

ANTMANN K, AMATO D, WOOD W, CORSON J, SUIT H, PROPPE K, CAREY R, GREENBERGER J, WILSON R AND FREI III, E. (1985). Selection bias in clinical trials. *J. Clin. Oncol.*, **3**, 1142–1147.

BAUM M. (1993). New approach for recruitment into randomised controlled trials. *Lancet*, **341**, 812–813.

CASSILETH BR, ZUPKIS RV, SUTTON-SMITH K AND MARCH V. (1980). Informed consent – why are its goals imperfectly realised? *N. Engl. J. Med.*, **302**, 896–900.

CULYER A. (1994). The Culyer Report.

DAVIS S, WRIGHT PW, SCHULMAN SF, HILL LD, PINKHAM RD, JOHNSON LP, JONES TW, KELLOGG Jr HB, RADKE HM, SIKKEMA WW, JOLLY PC AND HAMMAR SP. (1985). Participants in prospective, randomised clinical trials for resected non small cell lung cancer have improved survival compared with non participants in such trials. *Cancer*, **56**, 1710–1718.

EARLY BREAST CANCER TRIALISTS COLLABORATIVE GROUP. (1992). Systemic treatment of early breast cancer by hormonal, cytotoxic or immune therapy. 133 randomised trials involving 31 000 recurrences and 24 000 deaths among 75 000 women. *Lancet*. **339**, 1–15, 71–85.

FRIEDMAN MA AND CAIN DF. (1990). National Cancer Institute Sponsored Cooperative Clinical Trials. *Cancer*, **65**, (Suppl 10), 2376–2382.

GOTAY CC. (1991). Accrual to cancer clinical trials: directions from the research literature. *Soc. Sci. Med.*, **33**, 5, 569–577.

KARJALAINEN S AND PALVA I. (1989). Do treatment protocols improve end results? A study of survival of patients with multiple myeloma in Finland. *Br. Med. J.*, **229**, 1060–1072.

MACKILLOP WJ, PALMER MJ, O'SULLIVAN B, WARD GK, STEELE R AND DOTSIKAS G. (1989). Clinical trials in cancer: the role of surrogate patients in defining what constitutes an ethically acceptable clinical experiment. *Br. J. Cancer*, **59**, 388–395.

PENMAN DT, HOLLAND JC, BAHNA GF, MORROW G, SCHMALE AH, DEROGATIS LR, CARNRIKE Jr CL AND CHERRY R. (1984). Informed consent for investigational chemotherapy: patients' and physicians perceptions. *J. Clin. Oncol.*, **2**, 7, 849–855.

SIMES RJ, TATTERSALL MHN, COATES AS, RAGHAVEN D, SOLOMON HJ AND SMARTT H. (1986). Randomised comparison of procedures for obtaining informed consent in clinical trials of treatment for cancer. *Br. Med. J.*, **293**, 1065–1068.

SMYTH J, MOSSMAN J, HALL R, HEPBURN S, PINKERTON R, RICHARDS M, THATCHER N AND BOX J. (1994). Conducting clinical research in the new NHS: the model of cancer. *Br. Med. J.*, **309**, 457–461.

STILLER C. (1992). Survival of patients in clinical trials and at specialist centres. In *Introducing New Treatments for Cancer*, Williams CJ. (ed.) pp. 119–136. John Wiley: Chichester.

TANNOCK IF. (1992). Some problems related to the design and analysis of clinical trials. *Int. J. Radiat. Oncol. Biol. Phys.*, **22**, 881–885.

TAYLOR KM, MARGOLESE RG AND SOSKOLNE CL. (1984). Physicians' reasons for not entering eligible patients in a randomised clinical trial of surgery for breast cancer. *N. Engl. J. Med.*, **310**, 1363–1367.